# Application of Random Forest Survival Models to Increase Generalizability of Decision Trees: A Case Study in Acute Myocardial Infarction

**DOI:** 10.1155/2015/576413

**Published:** 2015-12-21

**Authors:** Iman Yosefian, Ehsan Mosa Farkhani, Mohammad Reza Baneshi

**Affiliations:** ^1^Regional Knowledge Hub and WHO Collaborating Center for HIV Surveillance, Institute for Futures Studies in Health, Kerman University of Medical Sciences, Kerman 7616911317, Iran; ^2^Department of Epidemiology, University of Tehran, Tehran, Iran; ^3^Research Center for Modeling in Health, Institute for Futures Studies in Health, Kerman University of Medical Sciences, Kerman 7616911317, Iran

## Abstract

*Background*. Tree models provide easily interpretable prognostic tool, but instable results. Two approaches to enhance the generalizability of the results are pruning and random survival forest (RSF). The aim of this study is to assess the generalizability of saturated tree (ST), pruned tree (PT), and RSF.* Methods*. Data of 607 patients was randomly divided into training and test set applying 10-fold cross-validation. Using training sets, all three models were applied. Using Log-Rank test, ST was constructed by searching for optimal cutoffs. PT was selected plotting error rate versus minimum sample size in terminal nodes. In construction of RSF, 1000 bootstrap samples were drawn from the training set.* C*-index and integrated Brier score (IBS) statistic were used to compare models.* Results*. ST provides the most overoptimized statistics. Mean difference between* C*-index in training and test set was 0.237. Corresponding figure in PT and RSF was 0.054 and 0.007. In terms of IBS, the difference was 0.136 in ST, 0.021 in PT, and 0.0003 in RSF.* Conclusion*. Pruning of tree and assessment of its performance of a test set partially improve the generalizability of decision trees. RSF provides results that are highly generalizable.

## 1. Introduction

The prediction of survival rate is a major aim in survival analysis. In the case of time-to-event data, Log-Rank test and Cox regression models are the most frequently used method. The Cox model can be used to identify the variables that significantly affect the outcome of interest and presents the results in terms of Hazard Ratio (HR) [1]. However, this model does not provide an easily interpretable decision rule to be used in clinical practice. In addition, exploration of presence of high order interactions needs inclusion of interaction terms in the model which makes the interpretation of results more difficult [2].

An alternative strategy which easily handles both these problems is decision tree analysis [3]. The trees consist of root, internal, or daughter nodes and terminal nodes. At the first step, all subjects are put in the root node. Subjects should be categorized into two daughter nodes with maximum difference between them. This will achieve by extensive search among all independent variables to find the variable (and cutoff) that maximizes the difference [4]. All possible cutoffs of all independent variables are tried to explore which one leads to the highest Log-Rank statistics (corresponding to the lowest *P* value). Once the first split is created, a similar approach is applied to each internal node. This leads to a tree structure which divides the subjects into the final terminal nodes [5–8]. These models provide pictorial decision rules and therefore can be easily used in medical decision making.

Once a model has been created some measures of model performance are required. For example, in the case of logistic regression, sensitivity and specificity, or area under ROC curve, should be reported. These statistics show how well the model discriminates between cases and controls.

In the case of survival analysis,* C*-index and Brier statistics are usually reported.* C*-index is a generalization of the area under ROC curve which compares survival rate of those who experienced the event with those who did not [9]. Brier score (BS) compares predicted survival rate with the actual status of patients [10]. High* C*-index and low BS indicate adequate fit of the model to the data.

In the process of model building, researchers usually fit a model using a given data set and then assess its performance using the same data set. Regardless of the method of model building, an important aim in risk prediction models is to construct models which accurately predicts the risk for future patients. It has been argued that use of a training set to construct the model and to assess its performance leads to overoptimized statistics with low generalizability [11]. The level of overoptimization in the case of decision tree models is even higher, due to extensive search at each node [12].

One of the easiest approaches to tackle the problem of overoptimized statistics is to randomly divide the data into training and test set. In this case, the model can be constructed on the training set. The model derived will then be applied on the test set to calculate the performance statistics [11]. This approach, however, leads to decrease in sample size and power.

Alternative approaches suggest bootstrap aggregation of the results [13, 14]. This means to construct the model on a number of randomly derived bootstrap samples (say 1000) and to test them using the same sample and to report the mean and standard deviation of the statistics of interest.

One of the aggregation methods which has been proposed is random survival forest models. This method controls for overoptimization by two mechanisms [15]. Firstly, it draws multiple bootstrap samples from the initial data. In addition to that, to construct each tree, a random sample of independent variables would be selected and used. It has been argued that using two forms of randomization in growing the trees and combination of them cause sensible reduction instability of a single tree. The objective of this study was to compare the performance of survival tree and random survival forest for predicting survival probability patients admitted with acute myocardial infarction.

## 2. Material and Methods

We used information of 607 acute myocardial infarction (AMI) patients aged >25 years, admitted to the CCU of Imam Reza Hospital Mashhad, Iran, in 2007. Patients were identified according to the International Classification of Diseases (ICD-10) with 12.0 to 12.9 codes. In the current study, the main outcome was death due to AMI. Time from admission to discharge or death was considered as follow-up time. Information of 11 predictor variables was as follows: age (in years), sex, hypertension disease (no and yes) (patients with systolic blood pressure ≥140 mmg or diastolic blood pressure ≥90 mmg were considered as “yes”), hyperlipidemia (no and yes), history of ischemic heart disease at admission (no and yes), diabetes (no and yes), smoking status (no and yes), family history of AMI disease, Q wave status (presence or absence of pathologic Q waves in electrocardiogram (ECG)), streptokinase treatment (no and yes), and intervention (angioplasty, pacemaker surgery, bypass surgery, and drug therapy).

We compared four methods as explained below: saturated survival tree, pruned survival tree, and Random Forest Survival (RFS) (see detail below). We randomly divided our data set into two parts, training and test sets, by using 10-fold cross-validation; then models were constructed using the training set. In saturated and pruned survival trees, performance was assessed on both training and test sets. In random survival forest, performance was assessed on out-of-bag and test sets (explained later).

### 2.1. Saturated Survival Tree

In construction of the survival tree, using training set, Log-Rank statistics was used as split criterion. A saturated tree was constructed under the restriction that a terminal node has at least 1 death. The performance of the final tree (in terms of IBS and* C*-index) was tested on both training and test samples.

### 2.2. Pruned Survival Tree

Secondly, the tree constructed using training sample was pruned. The tree size was plotted against error in test set (1 − *C* index) to select the optimal tree. Sampling variation was addressed as explained above.

### 2.3. Random Survival Forest

RSF is an ensemble method that introduces 2 forms of randomization into the tree growing process: bootstrap sampling from the data and selection of a limited number of independent variables to construct the tree [16].

Using the training set, RSF procedure was applied. Its performance was then assessed using OOB training and the test set. This procedure has been repeated 1000 times, as explained below.

First, an independent bootstrap sample is used for growing the tree. Second, to split each node of the tree into 2 daughter nodes, a limited number of covariates are selected. It has been shown that each sample would be selected in about 63% of samples. The samples not being selected are referred to as out-of-bag (OOB) sample. This means that, in 1000 bootstrap samples, each subject is a part of OOB 370 times. We followed the procedure below:

(1) 1000 bootstrap samples were drawn.
(2) In each sample, a survival tree was constructed. At each node of the tree, $\sqrt{p}$ candidate variables were selected. The node is split using the candidate variable that maximizes survival difference between daughter nodes.
(3) Based on the rules derived from trees, survival curves for OOB patients were plotted.
(4) For each subject, the average survival curves are calculated to be considered as subject's final $\hat{S}\left(t\right)$.

In all three approaches, 10-fold cross-validation was applied. To capture additional variations, the process of cross-validation was repeated 20 times, therefore creating 200 training and 200 test data sets at each method.

### 2.4. Performance Statistics

#### 2.4.1. *C*-Index

Let (*T*_1,*h*_, *σ*_1,*h*_), (*T*_2,*h*_, *σ*_2,*h*_),…, (*T*_*n*,*h*_, *σ*_*n*,*h*_) be the survival times and the censoring status for *n* subjects in a terminal node *h*. Also, let *t*_1,*h*_ < *t*_2,*h*_ < ⋯<*t*_*m*,*h*_ be the *m* distinct event times in terminal node *h*. Define *d*_*l*,*h*_ and *Y*_*l*,*h*_ to be the number of deaths and subjects at risk at time *t*_*l*,*h*_. The cumulative hazard function (CHF) estimate for terminal node *h* is the Nelson-Aalen estimator

(1) $$\begin{array}{c} {\hat{H}}_{h}\left(\;t\;\right)=\underset{t_{l,h}\leq t}{\sum }\frac{d_{l,h}}{Y_{l,h}} \end{array}$$

for the subject *i* with a *d*-dimensional covariate **x**_**i**_

(2) $$\begin{array}{c} H\left(\;t\;|\;x_{i}\;\right)={\hat{H}}_{h}\left(\;t\;\right),\;\text{if}\;\;x_{i}\in h. \end{array}$$

In RSF procedure, to estimate CHF of subject *i*, define *I*_*i*,*b*_ = 1 if *i* is an OOB case for *b*th bootstrap sample; otherwise, *I*_*i*,*b*_ = 0. Let *H*_*b*_〈*t*∣**x**_**i**_〉 denote the CHF for subject *i* in a tree grown from the *b*th bootstrap sample. The ensemble CHF for *i* is

(3) $$\begin{array}{c} H^{*}\left(\;t\;|\;x_{i}\;\right)=\frac{\sum_{b=1}^{B}I_{i,b}H_{b}\left(t\;|\;x_{i}\right)}{\sum_{b=1}^{B}I_{i,b}}. \end{array}$$

The* C*-index is calculated using the following steps:

(1) Form all possible pairs of subjects.
(2) Consider permissible pairs, by eliminating those pairs whose shorter survival time is censored, and by eliminating pairs (*i*, *j*) if *T*_*i*_ = *T*_*j*_ and both are deaths.
(3) For each permissible pair where *T*_*i*_ = *T*_*j*_, count 1 if the shorter survival time has high risk predicted; count 0.5 if risk predicted is tied. For each permissible pair, where *T*_*i*_ = *T*_*j*_ and both are deaths, count 1 if risk predicted is tied; otherwise, count 0.5. For each permissible pair where *T*_*i*_ = *T*_*j*_, but at least one is not a death, count 1 if the death has high risk predicted; otherwise, count 0.5. Let Concordance denote the sum over all permissible pairs.
(4) *C*-index = Concordance/permissible.

In the survival tree, we say *i* has a high risk predicted than *j* if

(4) $$\begin{array}{c} \overset{m}{\underset{l=1}{\sum }}H\left(\;t_{l}\;|\;x_{i}\;\right)>\overset{m}{\underset{l=1}{\sum }}H\left(\;t_{l}\;|\;x_{j}\;\right), \end{array}$$

where *t*_1_ < *t*_2_ < ⋯<*t*_*m*_ are the unique event times in the data set. In RSF ensemble CHF (*H* ^*∗*^(*t*∣**x**)) is used instead of *H*(*t*∣**x**) [16].

A value of 0.5 for* C*-index is not better than random guessing and a value of 1 denotes full-discriminative ability. Percentiles 2.5 and 97.5 were considered as lower and upper bounds of CI for final statistics.

#### 2.4.2. IBS Statistics

The Brier score at time *t* is given by
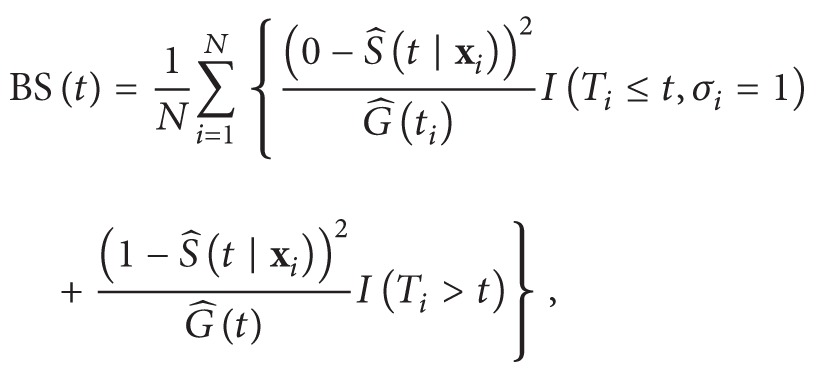
(5)where $\hat{G}\left(t\right)=P(C_{i}>t)$ denote the Kaplan-Meier estimate of the censoring survival function [17, 18].

The prediction error curve is gotten by calculating of Brier score across the times. In addition, the integrated Brier score (IBS) that is cumulative prediction error curves over time is given by

(6) $$\begin{array}{c} \text{IBS}=\frac{1}{\max\left(t_{i}\right)}\overset{\max\left(t_{i}\right)}{\underset{0}{\int }}\text{BS}\left(\;t\;\right)dt. \end{array}$$

Lower values of IBS indicate better predictive performances. Percentiles 2.5 and 97.5 were considered as lower and upper bounds of CI for final statistics.

### 2.5. Impact of Method of Tree Construction and Data Set on Performance Statistics

As explained above, three methods were applied to construct the tree (ST, PT, and RSF). In addition, two data sets (training and testing) were used to assess the performance. These two factors together created six scenarios with 200 replications in each. In each of 1200 samples, values IBS and* C*-index were recorded. Two way ANOVA was applied to assess the impact of method of tree construction and data used for validation on performance statistics.

### 2.6. Software

We used randomForestSRC and pec R-package for analyses of this study.

## 3. Results

Our data set comprised 607 patients with mean age of 61.34 years (SD = 13.46). In total, 204 patients experienced the outcome of interest (death due to AIM).  Table 1 provides information for the other 10 independent variables collected.

As summarized in Table 2, saturated tree provides the most overoptimized statistics in training set. While* C*-index in saturated tree was 0.872, corresponding figure for RSF is 0.710. In addition, difference of* C*-index in training and test sets in saturated tree was much higher than other methods (0.24 in saturated tree, 0.05 in pruned tree, and 0.006 in RSF).

Similarly, in saturated tree, estimation of IBS using training set provides results which were not replicated in test set (0.088 versus 0.224). Pruned tree partially tackles the problem. RSF provides the most comparable results.

### 3.1. Saturated Tree

**Figure 1:**
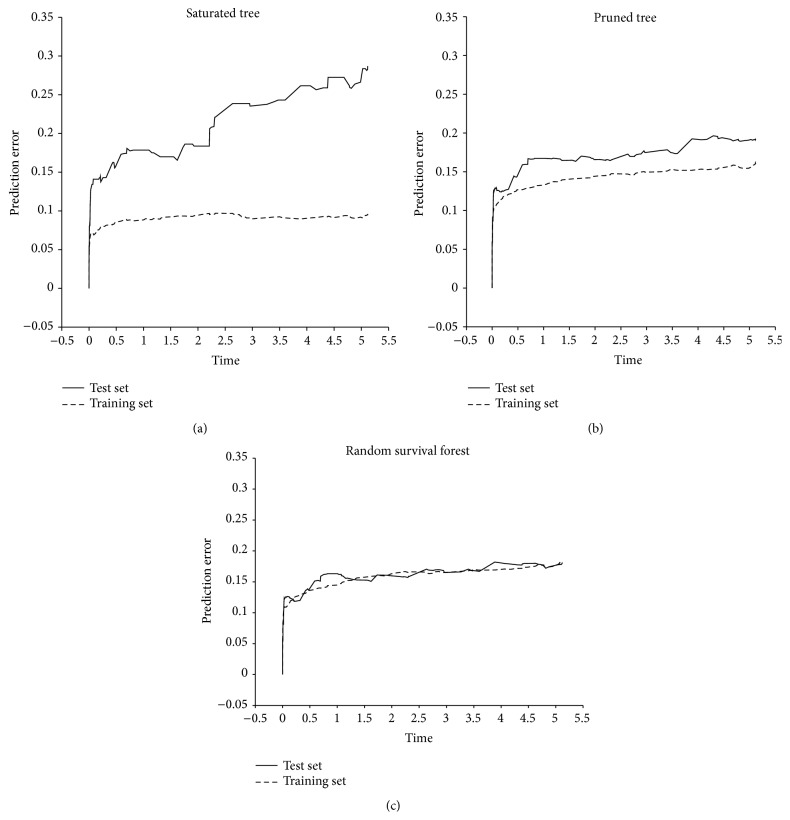
Comparison of Brier score (BS), over time, in training and test sets: (a) saturated tree, (b) pruned tree, and (c) random survival forest.

Once the saturated tree was applied to the training set, IBS was 0.088, indicating very low prediction error (Table 2). However, when this model was applied to the test set, IBS was increased by a factor of about 1.5 and reached to 0.224. In addition, about 27% reduction in* C*-index was seen. The* C*-index in training and test sets was 0.872 and 0.634, respectively. CIs suggested significant difference between these statistics in training and test sets.  Figure 1(a) shows BS values over time in training and test sets. BS in training set is consistently higher than the corresponding figure in test set.

### 3.2. Pruned Tree

Pruning the tree, still difference between performance on training and test sets was seen. However, the magnitude of the difference was much in comparison with saturated tree. Assessing the performance of pruned tree on the training set yields IBS of 0.145 (Table 2). Corresponding figure in test set was 0.166, corresponding to 17% increase.* C*-index values in training and test sets were 0.753 and 0.699, respectively. This indicates only 7% reduction. No significant difference between training and test sets was seen in terms of performance statistics. However, statistics corresponding to test set was much wider.  Figure 1(b) indicates that the difference between two lines (corresponding to training and test sets) is much lower than that of the saturated tree (Figure 1(a)).

### 3.3. RSF

In RSF performance on both training and test sets is approximately the same (Table 2). The IBS values were 0.163 and 0.163, respectively.* C*-index values were 0.710 and 0.716. Based on Figure 1(c), two lines cannot be distinguished. This indicates the high generalizability of RSF results. Similar to PT, performance statistics in training and test sets were not significantly different.

### 3.4. Impact of Method of Tree Construction and Validation Set on Performance Statistics

Both of these factors significantly affect the statistics. In addition, significant interaction between them was seen (all *P* values < 0.001):

(7) C-index=0.716−0.082Imodel = ST−0.017Imodel = PT−0.006Isample = Train+0.244Imodel = ST & sample = Train+0.060Imodel = PT & sample = Test,IBS=0.162+0.062Imodel = ST+0.004Imodel = PT+0.0003Isample = Train−0.137Imodel = ST & sample = Train−0.021Imodel = PT & sample = Test.

## 4. Discussion

Using an empirical data set, our results showed that assessment of performance of decision trees using training set led to huge overoptimized statistics. In particular, when a saturated tree was constructed difference between* C*-index in training and test set was as high as 0.24. Pruning the tree partially tackled the overoptimization where the difference was reached to 0.05. We should emphasize that 0.05 difference in* C*-index is considered as huge since this statistic varies between 0.50 and 1. On the other hand, RSF was the only method that provides comparable results, no matter which data set was used. Performance of methods in terms of IBS led to the same conclusion. Again RSF and saturated trees provided the most accurate and the most overoptimized statistics.

We have not implemented extensive simulation studies. However, similar manuscripts also suggested that RSF works better than other algorithms. Austin et al. used data of AMI patients data to compare performance of PT and RF. The main outcome was whether the patient died within 30 days of hospital admission. The number of independent variables was 33.* C*-index in training and test sets was 0.768 and 0.767, respectively. Applying RF, corresponding figures were 0.823 and 0.843. We guess that the closeness of results of PT, in training and test sets, was due to very large sample size in training set (9298) [19].

Opitz and Maclin used 23 data sets from University of Wisconsin Machine Learning Repository (UCI data) to compare bootstrap aggregated and pruned trees. Performance of models was checked using 10-fold cross-validation. In all data sets, error rates corresponding to bagged trees were lower than pruned trees [20].

Walschaerts et al. used data of 144 breast cancer patients to compare PT and RSF. Data was randomly divided into training and test sets 30 times. Models were constructed on training and its performance was checked on test sets. The number of independent variables was 75: five clinical risk factors and 70 gene expression measurements. Mean error rate (1 −* C*-index) in PT and RSF was 0.389 and 0.279, respectively [21].

Bou-Hamad et al. used information of 312 patients who suffered from primary biliary cirrhosis of the liver. The number of independent variables was 12. They compared Cox regression, PT, bagging trees, and RSF in terms of IBS. 10-fold cross-validation was applied to assess the performance of models. Results were presented graphically and suggested that RSF provides the best results, followed by bagging. PT provided the poorest results. The performance of the Cox regression model was in between [22].

As expected, results from literature and ours indicate higher generalizability of ensemble methods such as RSF. One of the strengths of our study is that we compared 3 different approaches on both training and test sets. We also calculated* C*-index and IBS statistics to compare the performance of different approaches. Majority of articles only compared pruned tree with RSF using test sets.

One of the limitations of our study was that we were not able to plot the mean of prediction error curve oversamples. We simply selected one of randomly generated samples to monitor the trend of BS over time. However, we reported the mean values to take into account the sampling variations. In addition, in our empirical data set the Event Per Variable (EPV) was about 20. We expect poorer performance for saturated and pruned trees at low EPVs. The impact of EPV on performance of alternative methods remains to be addressed.

## 5. Conclusion

We do recommend use of a training set to assess the performance of statistical models including decision trees. Pruning of tree partially tackles the degree of overoptimization. However, still high difference between training and test sets is expected. On the other hand, RSF provides statistics which can be generalized to independent samples.

## Figures and Tables

**Table 1:** Demographic characteristics of patients.

Predictor variables	Levels	Number (percent%)
Sex	Male/female	423 (69.7)/184 (30.3)
Hypertension disease	Yes/no	245 (40.4)/362 (59.6)
Hyperlipidemia	Yes/no	135 (22.2)/472 (77.8)
History of ischemic heart disease	Yes/no	184 (30.3)/423 (69.7)
Diabetes	Yes/no	150 (24.7)/457 (75.3)
Smoking status	Yes/no	216 (35.6)/391 (64.4)
Family history of AMI disease	Yes/no	63 (10.4)/544 (89.6)
Q wave status	Yes/no	159 (26.2)/448 (73.8)
Streptokinase treatment	Yes/no	278 (45.8)/329 (54.2)
Intervention	Angioplasty	32 (5.3)
	Pacemaker surgery	36 (5.9)
	Bypass surgery	45 (7.4)
	Drug therapy	494 (81.4)

**Table 2:** Assessment of performance of different tree construction methods using either training or test sets.

	*C*-index			IBS		
	Training set	Test set	Percent change	Training set	Test set	Percent change
Saturated tree	0.872 (0.863, 0.882)	0.634 (0.528, 0.743)	27%	0.088 (0.082, 0.094)	0.224 (0.157, 0.298)	150%
Pruned tree	0.753 (0.740, 0.768)	0.699 (0.570, 0.824)	7%	0.145 (0.138, 0.151)	0.166 (0.113, 0.221)	14%
RSF	0.710 (0.693, 0.729)	0.716 (0.609, 0.857)	0.08%	0.163 (0.156, 0.169)	0.163 (0.114, 0.210)	0.1%
